# Chinese Herbal Formula Xiao Yao San for Treatment of Depression: A Systematic Review of Randomized Controlled Trials

**DOI:** 10.1155/2012/931636

**Published:** 2011-08-22

**Authors:** Yuqing Zhang, Mei Han, Zhijun Liu, Jie Wang, Qingyong He, Jianping Liu

**Affiliations:** ^1^Guang'anmen Hospital, China Academy of Chinese Medical Science, Beijing 100053, China; ^2^Centre for Evidence-Based Chinese Medicine, Beijing University of Chinese Medicine, Beijing 100029, China

## Abstract

*Objectives.* To assess the beneficial and adverse effects of Xiaoyaosan for depression. *Search Strategy*. Electronic databases were searched until December 2009. *Inclusion Criteria.* We included randomized clinical trials testing Xiaoyaosan against placebo, antidepressants, or combined with antidepressants against antidepressants alone. *Data Extraction and Analyses.* Study selection, data extraction, quality assessment, and data analyses were conducted according to the Cochrane standards. *Results.* 26 randomized trials (involving 1837 patients) were included and the methodological quality was evaluated as generally low. The pooled results showed that Xiaoyaosan combined with antidepressants was more effective in comprehensive effect, the score of HAMD and the score of SDS compared with antidepressants alone. Xiaoyaosan was superior to antidepressants for the score of HAMD. However, Xiaoyaosan was not different from placebo for the score of SDS. There was no adverse effects reported in the trials from Xiaoyaosan. *Conclusions.* Xiaoyaosan appears to be effective on improving symptoms in patients with depression. However, due to poor methodological quality in the majority of included trials, the potential benefit from Xiaoyaosan need to be confirmed in rigorous trials and the design and reporting of trials should follow international standards.

## 1. Introduction

Depression is a common mental disorder that presents with depressed mood, loss of interest or pleasure, feelings of guilt or low self-worth, disturbed sleep or appetite, insomnia or hypersomnia, fatigue or loss of energy, and poor concentration or difficulty making decisions. These problems can become chronic or recurrent and lead to substantial impairments in an individual's ability to take care of his or her everyday responsibilities. 

Depression is recognized as a major public health problem, which has a substantial impact on individuals and society. Depressive disorders are common in the general population. It affecting about 121 million people worldwide. At its worst, depression can lead to suicide, a tragic fatality associated with the loss of about 850 000 thousand lives every year. Depression is the leading cause of disability as measured by Years Lived with Disability (YLD). The World Health Organization has described depression as an “unseen burden” [1, 2]. It was the 4th leading contributor to the global burden of disease when measured by Disability Adjusted Life Years (DALYs) in 2000. By the year 2020, depression is projected to reach 2nd place of the ranking of DALYs calculated for all ages in both sexes. Today, depression is already the 2nd cause of DALYs in the age category 15–44 years for both sexes combined. Mood disorders have been shown to have a greater impact on quality of life compared with conditions such as hypertension and cardiac disease [3].

In China, depression is now one of the top three public health problems. Statistics show that 5 per cent of Chinese people suffer from the disease and 13 out of 1,000 Chinese have mental health issues [4]. Until 2003, China has over 26 million depression patients, for which discrimination and neglect are the two major obstacles to curing them, thus incurring an annual loss of over 64 billion yuan, according to a reserved estimation made at the Seminar on Attention to the Socio-Economic Burden of Depression held [5].

Depression is most commonly treated with antidepressants in primary care [6]. In addition, there are other kind of psychological interventions, such as cognitive behavior therapies, interpersonal therapy, psychotherapy, and counseling. The most widely prescribed antidepressants come from a class of medications known as selective serotonin reuptake inhibitors (SSRIs). Besides of that “atypical” antidepressants, the older tricyclic antidepressants and monoamine oxidase inhibitors (MAOIs). The common side effects include sexual problems, drowsiness, fatigue, sleep difficulties, nausea weight gain, nervousness, dry mouth, and blurred vision. Stop taking drugs abruptly may cause “antidepressant discontinuation syndrome” such as spells, extreme restlessness, dizziness, fatigue, and aches and pains.

The Xiaoyaosan (XYS) decoction containing eight commonly used herbs (Bupleurum root, Chinese angelica root, white peony root, poria, bighead atractylodes rhizome, roasted ginger, prepared licorice root, menthol and peppermin) been used for treatment of mental disorders such as depression for centuries in China. The mechanism of the description maybe soothing the liver, invigorating the spleen, nourishing the blood to restore the normal menstruation, and clearing away the liver fire due to blood deficiency. Biochemically, the XYS decoction also reversed CIS-induced decreases in brain-derived neurotrophic factor (BDNF) and increases in tyroxine hydroxylase (TrkB), and neurotrophin 3 (NT-3) in the frontal cortex, and the hippocampal CA subregion. 

Currently, xiaoyaosan used alone or integrated with antidepressants has been widely used as an alternative and effective method for the treatment of depression in China. Many clinical studies reported the effectiveness ranging from case reports and case series to controlled observational studies and randomized clinical trials, but the evidence for its effect is not clear. The present paper aims to evaluate the beneficial and harmful effects of xiaoyaosan (wan) for treatment of depression in randomized trials.

## 2. Methods

### 2.1. Database and Search Strategies

Literature searches were conducted in National Knowledge Infrastructure (1999–2009), VIP Database for Chinese Technical Periodicals (1989–2009), Chinese Biomedical Literature Database (1995–2009), PubMed (1950–2009), and Cochrane library (Issue 3, 2009) and searched the reference list of retrieved papers. All of those searches ended the end of November 2009. We used the search terms “depression,” “xiaoyaowan,” “xiaoyaosan,” and “xiaoyaotang.” Various combinations of the terms were used, depending on the database searched. The bibliographies of included studies were searched for additional references.

### 2.2. Inclusion Criteria

All the parallel randomized controlled trials (RCTs) of all the prescriptions based on “xiaoyaosan” including pills, powder, decoction dosage form compared with antidepressants in patients with depression were included. RCTs combined xiaoyansan with antidepressants compared with antidepressants and all the modified xiaoyaosan formula were included as well. There were no restrictions on population characteristics, language and publication type. 

Outcome measures include *Clinical Comprehensive Effect*, Hamilton depression scale (HAMD) scores, self-rating depression scale (SDS) scores, self-rating anxiety scale (SAS) scores, Hamilton Anxiety Scale (HAMA), scores, clinical global impression (CGI) scores, the scale for TCM syndrome and symptom differentiation (TCM-SSD) scores and so forth, the criteria “recover, significant effective, effective, or not effective” was also include in the outcome measurement. Duplicated publications reporting the same groups of participants were excluded.

### 2.3. Data Extraction and Quality Assessment

Two authors (J. P. Liu and Y. Q. Zhang) extracted the data from the included trials independently. The methodological quality of trials was assessed using the 6 criteria 6 election bias (study design, confounders, blinding, data collection methods, withdrawals and dropouts) to following 3 categories: Category A (strong quality): four strong ratings with no weak ratings above. Category B (moderate quality): less than four strong ratings and one weak rating. Category C (weak quality): two or more weak ratings. 

Quality assessment of included randomized controlled trials: sequence generation, allocation concealment, blinding of participants personnel and outcome assessors, incomplete outcome data, selective outcome reporting, and other sources of bias.

### 2.4. Data Synthesis

The statistical package (RevMan 4.3.2) was used for data analyses, which was provided by The Cochrane Collaboration. Dichotomous data were presented as risk ratio (RR) and continuous outcomes as mean difference (MD), both with 95% confidence interval (CI). Meta-analysis was performed if the intervention, control, and outcome were the same or similar. The statistical heterogeneity was presented as significant when *I* square (*I* ^2^) is over 50% or *P* < 0.1. Random effect model was used for the meta-analysis if there was significant heterogeneity (*I* ^2^ > 50%) and fixed effect model was used when the heterogeneity was not significant (*I* ^2^ < 50%) [7].

## 3. Result

### 3.1. Description of Included Trials

**Figure 1:**
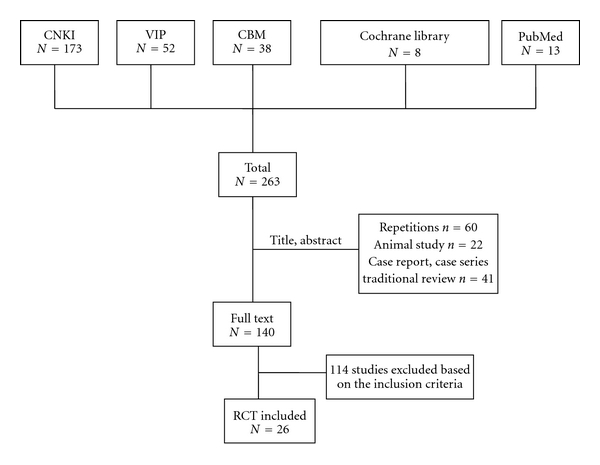
Study selection process.

After primary search of 5 databases, 263 trials were screen out from electronic and manual searches (Figure 1), and the majority were excluded due to obvious ineligibility which including irrelevant titles and abstract (some papers being found from more than one database). 141 trials with full text papers were retrieved. 122 RCTs were excluded because of reporting the depression complicated with other disease such as stroke and postpartum, 60 trials were excluded because of duplicated publication, 22 trials were excluded due to the animal studies, and the rest 41 trials were noncontrolled clinical trials including case report, case series traditional review. 114 out of the rest of 140 articles were excluded based on the inclusion criteria. The treatment for depressive neurosis, bipolar disorders, and depression in patients with psychological stress insomnia were excluded. In the end, 26 RCTs were reviewed. The characteristics of 26 randomized trials are summarized in Table 1. 

The 26 RCTs involved 1837 patients with depression. There was a wide variation in the age of subjects (17–80 years). Twenty-six (26) trials specified six diagnostic criteria, four trials [7, 17, 22, 25, 31, 32] used the China classification and diagnostic criteria for mental disorder (second edition CCMD-2-R) alone, six [8, 9, 11, 18, 20, 24, 26–28, 30] trials used the third edition (CCMD-3) alone, three trials combined international classification on the diagnosis of depression (ICD-10) with CCMD-2-R [19] or CCMD-3 [12, 15], one trials [21] combined the depression standard in “internal medicine of Chinese medicine” and CCMD-3 together, one trial [16] used CCMD-3 and affective disorder in the western medicine and Chinese medicine classification and diagnostic criteria on depression breaks out, two trials [10, 29] used CCMD-3 and the diagnostic criteria of Chinese medicine on depression and stagnation of liver qi, one trial [23] used CCMD-3 and depression of liver-qi stagnation and spleen deficiency, the last trial [13] used the depression standard in TCM on liver-qi stagnation and spleen deficiency.

The interventions included all the prescriptions based on “xiaoyaosan” including pills, powder, decoction dosage form alone, with Maprotiline placebo or with antidepressants. The controls included antidepressants alone or the combination of danzhiXiaoyaosan (DXS) placebo and antidepressants. Eight trials investigated the prescriptions based on “xiaoyaosan” using alone [9, 17–19, 23, 30] or plus placebo [12, 15] versus antidepressants, one three-arm trial and the rest sixteen trials [7, 8, 10, 11, 13, 14, 16, 20–22, 24–29, 31, 32] compared the prescriptions based on “xiaoyaosan” plus antidepressants versus antidepressants.

The total treatment duration ranged from 30 days to 3 month. The variable prescriptions are presented in Table 1. The different composition of formula Xiaoyaosan are presented in Table 2. Nineteen (19) of the 26 trials used the hamilton depression scale (HAMD) as the outcome measure, other 4 kinds of scales including self-rating depression scale (SDS), self-rating anxiety scale (SAS), the scale for TCM syndrome and symptom differentiation (TCM-SSD), the hamilton anxiety scale (HAMA) were also be used. Side effect was evaluated by asberg side effect scale and treatment-emergent symptom side effect (TESS) scale or described in details. Eleven (11) trials used four classes to evaluate treatment effects including cure, significant effective, effective, ineffective, while ten (10) trials used three classes (except of cure) according to the scores reducing rate.

### 3.2. Methodological Quality of Included Trials

Six [10, 12, 15, 16, 23, 26] out of 26 trials (23.08%) were evaluated as strong quality, the rest of 20 trials (76.928%) were evaluated as moderate quality. The majority of the included trials were assessed to be moderate methodological quality. The sample size of including trials varied from 24 to 200 patients. None of the 26 trials reported sample size calculation. Ten trials described the randomization procedure, six [10, 12, 15, 16, 23, 26] trials used random number table, four trials [8, 17, 25, 30] used visiting time to realize the randomization. One trial [23] used opaque envelopes to allocate concealment. Only four [12, 15, 23, 27] of the 26 trials employed a blinding procedure: three of them using patients blinding, doctors blinding, and assessors blinding, and the other one [27] mentioned single-blind without further details. Seven trials [8, 10–12, 15, 21, 25] reported the withdrawals/dropouts information. Three trials [13, 16, 26] mentioned follow-up, and neither of them used intention to treat method. The reporting quality of 26 trials according to quality assessment tool for quantitative studies varied among different trials (Table 3).

### 3.3. Effect of the Interventions (Tables 4–6) 

#### 3.3.1. “Xiaoyaosan” versus Antidepressants (Western Medicine)

Eight [9, 12, 15, 17–19, 23, 30] trials compared xiaoyaosan with antidepressants.

**Clinical Comprehensive Effect**

Seven of the eight trials used clinical comprehensive effect to evaluate the outcome. Five trials [9, 15, 18, 23, 30] used the percentage of HAMD scores reduced rate to measure the outcome: cure (HAMD scores reduced rate more than 75%), significant effective (HAMD scores reduced rate between 51% and 75%), effective (HAMD scores reduced rate from 25 to 50%) and ineffective (HAMD scores reduced rate less than 25%). None of the five trials showed significant difference between treatment and control group on the four criteria outcome measurement Two [17, 19] trials compared the effectiveness using the three criteria outcome measurement: significant effective (SDS scores reduced rate ≥ 50%), effective (50 > SDS scores reduced rate ≥ 30%), not effective (SDS scores reduced rate < 30%). The Total effective rate is the combination of “cure”, “significant effective “and” effective rate”. We put these two different kinds of measurements together to evaluate the general effectiveness. The meta-analysis showed no significant difference between xiaoyaosan and antidepressants on the Total effective rate (RR: 1.05 [1.00, 1.11]; *P* = 0.07) (Table 4).

**HAMD Scores Decrease**

Meanwhile four trials [12, 15, 18, 30] reported there are no significant difference on the HAMD scores decrease nor on the HAMD scores reduced rate [15] after 6 weeks treatment. Meta-analysis of three trials showed the same result in the fixed effects model (WMD: 0.59 [−0.51, 1.70]; *P* = 0.29) and random effects model (WMD: 0.43 [−2.14, 2.99]; *P* = 0.74) with significant heterogeneity (*I* ^2^ = 68.7%) (Table 5).

**SDS Scores Decrease**

Four trails [15, 17, 19, 30] reported the SDS scores decreasing. Meta-analysis of four trials showed significant difference in favor of modified xiaoyao powder or decoction compare to antidepressants (WMD: −3.97 [−5.52, −2.41]; *P* < 0.00001) (Table 6).

**Other Outcomes (TCM-SSD Scores, SAS Scores, 5-HT, BDNF, etc.)**

One trial [15] showed that there are no significant differences on TCM-SSD and SAS scores. One trial [12] showed that after 6 weeks of treatment,the serum level of 5-hydroxytryptamine (5-HT) and brain-derived neurotrophic factor (BDNF) increased (*P* < 0.01) and the Interleukin-6 (IL-6) level decreased in both groups without significant difference between two groups, the cortisol (CORT) level reduced significantly in the DXP group compared to Maprotiline group.

#### 3.3.2. “Xiaoyaosan” Plus Antidepressants versus Antidepressants

Seventeen trials [7, 10, 11, 13, 14, 16, 20–22, 24–29, 31, 32] compared the combination of xiaoyaosan or modified xiaoyaosan plus antidepressants with antidepressants.

**Clinical Comprehensive Effect**

Meta-analysis of fourteen trials showed significant difference in favor of combination group on clinical comprehensive effect (RR: 1.10 [1.04, 1.17]; *P* = 0.0007) (Table 4).

**HAMD Scores Decrease**

Fourteen trials reported the HAMD scores decrease. The meta-analysis of fifteen trials showed there are significant beneficial effect on the combination group compare to the antidepressants using alone both in the fixed effects model (WMD: −0.51 [−0.71, −0.31]; *P* < 0.0001) and random effects model (WMD: −0.69 [−1.25, −0.13]; *P* = 0.02) with significant heterogeneity (*I* ^2^ = 76.3%).

**SDS Scores Decrease**

One trial [13] showed significant benefit on SDS scores decreased in favor of combination group after 6 (MD: −3.6 [−4.65, −2.55]; *P* < 0.00001) weeks treatment (Table 6).

**Other Outcomes (HAMA Scores, CGI Scores)**

One trial [10] showed significant benefit on HAMA scores decreased in favor of combination group after 6 (MD: −2.4 [−4.23, −0.57]; *P* = 0.01) weeks treatment.

One trial [10] reported the outcome of CGI scores. It used modified DanzhiXiaoyao decoction plus fluoxetine versus fluoxetine showed better effect on the combination group (MD: 0.8 [−1.2, −0.4]; *P* < 0.0001).

### 3.4. Publication Bias

**Figure 2:**
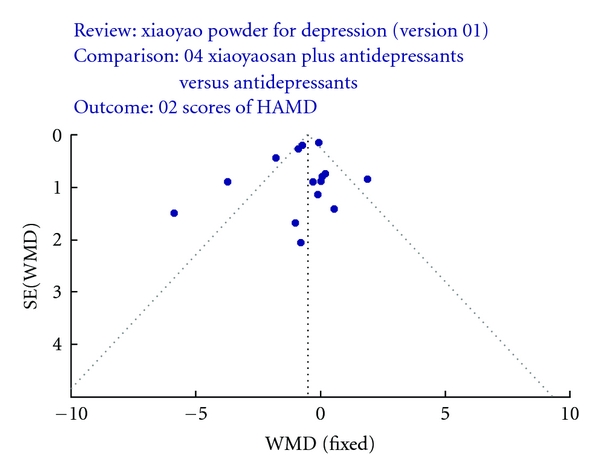
Funnel plot.

A funnel plot analysis of the 14 trials comparing xiaoyaosan plus antidepressants to antidepressants on Clinical Comprehensive Effect was generated, and it showed a significant asymmetry (Figure 2).

### 3.5. Adverse Effect

**Figure 3:**
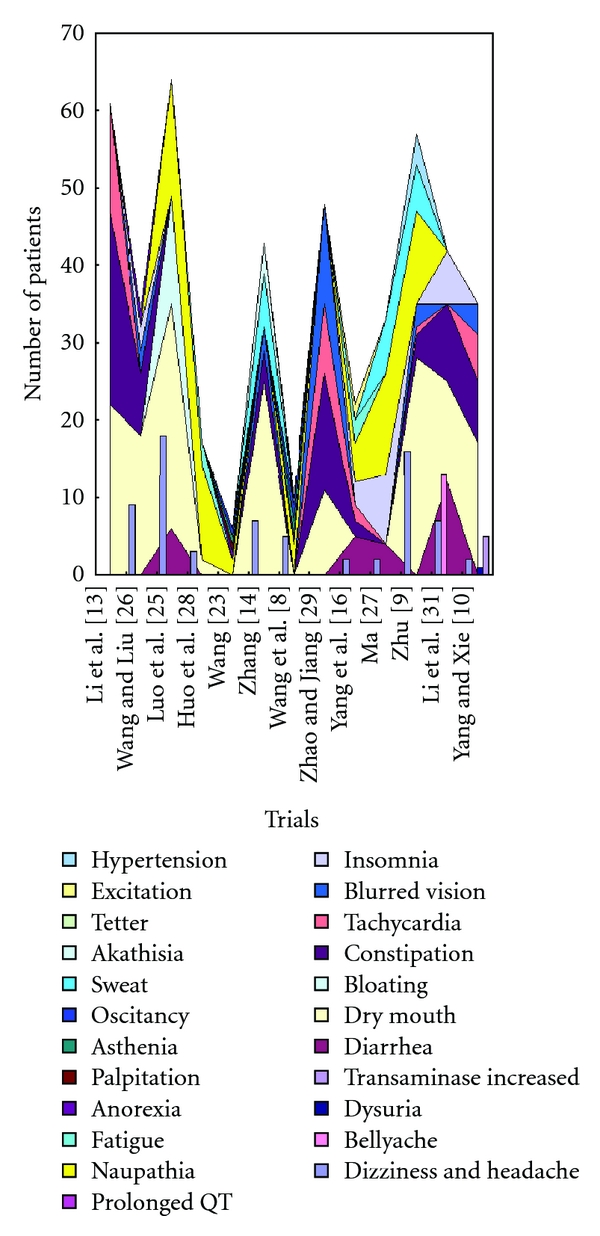
Side effect of including studies (“xiaoyaosan” versus antidepressants).

Twenty-four out of twenty-six trials mentioned the adverse effect except two trials [22, 24]. Twenty- four trials reported the twenty-seven specific symptoms including diarrhea, dizziness and headache, somnolence, dry mouth, Bloating, constipation, tachycardia, blurred vision, insomnia, prolonged QT, naupathia, fatigue, anxiety, tremor, anorexia, palpitation, asthenia, oscitancy, sweat, akathisia, tetter, excitation, hypertension, bellyache, dysuria, transaminase increased, and sexual dysfunction (Figure 3). 

Amitriptyline showed main side effect including dry mouth, constipation, dizziness, blurred vision, tachycardia, somnolence and so forth, [8, 9, 14, 18, 22, 25, 32]. Imipramine, chlorimipramine, doxepin showed main side effect including dry mouth, constipation and other symptoms in alimentary canal [11]. Fluoxetine, paroxetine, citalopram showed main side effect including anxiety, insomnia, headache, naupathia, sexual dysfunction, and tremor, [8, 13, 16, 19–21, 23, 26–28, 30]. Venlafaxine showed main side effect including dry mouth, sweat, insomnia, headache, and anxiety, [17, 31].

Four trials [9, 17, 19, 23] reported no side effect in the herbal medicine group compared to the antidepressants group. Three trials reported side effect in xiaoyaosan group including headache, dizziness, and slightly diarrhea [15, 18, 30]. Fifteen out of eighteen trials reported the combination group has less side effect compare to the antidepressants group. Twelve trials [7, 15–19, 21, 25, 29–31] mentioned the side effect are significant reduced in intervention group compared to control group. Seven trials [7, 16, 18, 21, 25, 30, 31] used treatment-emergent symptom side effect (TESS) scale scores, one trial [15] used asberg side effect scale scores, the rest three trials [17, 19, 29] did not mentioned the tools they used to evaluate the side effect. 

A meta-analysis of four trials [7, 8, 16, 27] with five comparison using TESS scale scores showed less side effect (WMD: −2.51 [−3.18, −1.84]; *P* < 0.00001) using xiaoyaosan plus antidepressants compare to antidepressants using alone with significant heterogeneity (*I* ^2^ = 87.2%). There is another trial [18] showed modified xiaoyao decoction had less side effect compared to amitriptyline (WMD: −1.86 [−2.57, −1.15]; *P* < 0.00001).

## 4. Discussion

Based on this paper and meta-analyses of the outcome on Clinical Comprehensive Effect, HAMD scores, SDS scores, HAMA scores, and CGI scores, the prescriptions based on “xiaoyaosan” including pills, powder, decoction dosage form using alone or combined with antidepressants may have beneficial effects on patients with depression. The prescription xiaoyaosan may have the same effectiveness as antidepressants at the end point of the treatment with fewer side effects. The combination group may have significant beneficial effect compared to the antidepressants group variable on onset time with less side adverse events. We tried to analysis the trend of “xiaoyaosan”'s effectiveness by different followup time points as well.

The SAS scales scores, TCM-SSD scales scores and the outcome of the four criteria outcome measurement “cure, significant effective, effective, or ineffective” showed that there are no significant differences between the prescription group and antidepressants group. Meanwhile the xiaoyaosan prescriptions [12, 15] using alone may not as effective as antidepressants after 2 weeks treatment but after 4 or 6 weeks treatment the effectiveness tend to be no significant difference between two groups. We could clearly tell the trend from the HAMD scores and the reduced rate [15] of HAMD scores [12, 15]. The SDS scores showed the xiaoyansan prescriptions are significantly more effective after 4 weeks [17] and 6 weeks [19] treatment compared to antidepressants.

The combination of xiaoyaosan prescription plus antidepressants group may have significant beneficial effect compared to the antidepressants group. The onset time are variable may depended on the form of prescription such as pills and decoctions. 

Two meta-analysis on HAMD scores showed significant heterogeneity. It may due to the different intervention and treatment time or the methodology quality. The significant heterogeneity on TESS scales may due to the dosage of the antidepressants.

According to the twenty-six trials the xiaoyansan prescription group and the combination of xiaoyaosan and antidepressants group have less adverse events compared to antidepressants group with significant differences which were showed by the TESS scales and Asberg side effect scales.

We should consider several limitations before accepting the findings of this paper. First, the quality of the included studies is generally moderate according to the quality assessment tool for quantitative Studies (Effective Public Health Practice Project 2007) which was recommended on the Cochrane Handbook. It also indicated that there are moderate risk of bias in most of the trials. Due to inadequate reporting of the allocation sequence, allocation concealment, blinding, intention to treat analysis and drop outs account in the majority of trials, it was possible that there was performance bias and detection bias due to patients and researchers being aware of the therapeutic interventions for the subjective outcome measures. Most of the trials provided limited descriptions of study design, randomization were mentioned but without further details after randomly assignment of patients which do not allow a proper judgment of the conduct of the trials. Therefore, we canot draw a confident conclusion that there are significant beneficial effects in patients with depression on combined groups or xiaoyaosan prescriptions using alone comparing to antidepressants. The number of trials identified limits us to perform meaningful subgroup or sensitivity analyses to illuminate robustness of the results in the review. Sixteen out of twenty-seven trials didnot described the blinding in details, only two trials [12, 15] used double-dummy in their study design.

Second, Liu et al. [33] found that some Asian countries including China publish unusually high proportions of positive results, considering all of the nineteen trials included are in Chinese the publication bias possibly existed. We cannot explore quantitatively the possibility of publication bias due to the small number of trials.

Third, different modified xiaoyao prescriptions and different form of the prescriptions were used in the trials: eight trials [7, 8, 11, 16, 20, 27, 28, 31] used fixed xiaoyaowan throughout the treatment, five trials used modified xiaoyaowan, and one trial used modified xiaoyansan based on menstruation period of young female [9]. The rest thirteen trials [10, 13, 14, 18, 19, 21–24, 29, 32] used modified xiaoyao decoction according to syndrome differentiation based on Chinese medicine theory, the herbal compound varied from 7 to 17 herbs (Table 2). The treatment duration varied from 30 days to 3 months.

Fourth, the use of composite outcome measures in 26 trials to evaluate overall improvement of symptoms limits the generalization of the findings. The classification of cure, significant effective, effective, or ineffective and the Total effective rate are not internationally recognized, and these outcome measurement are vague to interpret the effect. We suggest future trials to comply with international standards in the evaluation of treatment effect.

Most of the sample size in the including 26 trials is small and there is a moderate risk of bias. Further high-quality studies with larger sample size are needed to confirm the effectiveness of xiaoyaosan in treating depression. Proper randomization techniques need to be clearly described and fully reported. Blinding and double-dummy should be used and reported clearly although the double-dummy of the herbal decoction might be very difficult, blinding of patients and outcome assessors should be used to minimize performance and assessment biases. Intention-to-treat principle and appropriate method for including drop out into data analyses are also important in the design of the trials. Since different forms of xiaoyaosan prescriptions were used in patients with depression such as pills and decoctions, they are likely to have different onset time according to the existed trials. Therefore, future clinical trials may focus on particular subgroups or large sample size to evaluate the effect of different forms of xiaoyaosan prescriptions on treating patients with depression. Further randomized trials with well design and adequate sample size are warranted to support or refute the positive findings. Trials should be reported according to the CONSORT Statement [34]. 

In general, comparing to three categories (tricyclic-tertiary amines, nontricyclic, specific serotonin reuptake inhibitors (SSRIs)) of antidepressant drugs such as Amitriptyline, venlafaxine and Fluoxetine, the prescription based on xiaoyaosan in different forms appears to improve the symptoms with less adverse event. The combination of xiaoyaosan and antidepressants may have shorter onset time compare to antidepressants using alone. The mechanism [12] may due to the regulating the levels of 5-HT, CORT, BDNF, IL-6. Since depression may occurred recurrently with or without treatment, a longer follow-up period with serial measurement of outcomes after the treatment is important to determine the effectiveness and long term effect of the xiaoyaosan prescription. Considering there are not sufficient amount of high-quality trials on xiaoyaosan prescription treating patients with depression, the effectiveness of xiaoyaosan prescription need further rigorous trials to prove.

##  Conflict of Interest

The authors declare that there is no conflict of interest.

## Figures and Tables

**Table 1:** Characteristics and methodological quality of included studies.

Study ID	Sample	Diagnosis standard	Intervention	Control	Course (week)	Followup (month)	Outcome measure
Du et al. [8]	120	CCMD-3	xiaoyao pill plus fluoxetine	amitriptyline paroxetine	6–8		HAMD and TESS score, side effect
Huang and Gan [9]	69	ICD-10	Shugan jieyu formula	amitriptyline	6		clinical effect, side effect
Huo et al. [10]	72	CCMD-3	danzhi xiaoyao decoction plus fluoxetine	fluoxetine	6		clinical effect, HAMD, HAMA and CJI score, side effect
J. Li et al. [11]	60	CCMD-3	xiaoyao pill plus imipramine	imipramine	8		clinical effect, HAMD score, side effect
Y. J. Li et al. [12]	66	CCMD-3, ICD-10	danzhixiaoyao powder	maprotiline	6		HAMD score, 5-HT, NE, EDNF, CORT, IL-6, IL-1*β*
H. Li et al. [13]	80	Unclear	xiaoyao powder plus fluoxetine	fluoxetine	6	12	clinical effect, HAMD, SDS score, side effect
Liu and Chen 2007 [14]	78	“practice of internal medicine”-3	danzhixiaoyao powder plus amitriptyline	amitriptyline	8		clinical effect, HAMD score
Luo et al. 2006 [15]	66	CCMD-3, ICD-10	danzhixiaoyao powder	maprotiline	6		symptoms, clinical effect, HAMD, SDS, SAS and Asberg score, side effect
Ma 2007 [16]	60	CCMD-3	xiaoyao pill plus citalopram	citalopram	8	12	clinical effect, HAMD and Tess score, side effect
C. Y. Wang et al. 2004 [17]	200	CCMD-2-R	guipi xiaoyao pill	venlafaxine	4		clinical effect, SDS score, side effect
R. C. Wang 2008 [18]	60	CCMD-3	modifiedxiaoyao powder	amitriptyline	6		clinical effect, HAMD score, side effect
T. Y. Wang 2001 [19]	61	CCMD-2-R ICD-10	danzhixiaoyao decoction	fluoxetine	6		clinical effect, SDS score, side effect
W. A. Wang et al. 2005 [20]	68	CCMD-3	xiaoyao pill plus doxepin	fluoxetine	6		clinical effect, HAMD and HAMA score, side effect
Y. Wang and Liu [21]	60	CCMD-3	danzhixiaoyao powder plus fluoxetine	fluoxetine	8		clinical effect, HAMD and CGI-SI score, side effect
Wei et al. 1999 [22]	60	CCMD-2-R	xiaoyao powder plus amitriptyline	amitriptyline	4		clinical effect
Xian et al. [23]	60	CCMD-3	xiaoyao powder	fluoxetine	6		symptoms, clinical effect, HAMD score
Xiao [24]	66	CCMD-3	danzhixiaoyao powder plus clomipramine	clomipramine	8		clinical effect
Yang and Xie [25]	58	CCMD-2	xiaoyao powder plus amitriptyline or clomipramine	amitriptyline or clomipramine	12		clinical effect, TESS score, side effect
Yang et al. [26]	64	CCMD-3	modified xiaoyao pill plus amitriptyline	fluoxetine	12	3	clinical effect, HAMD score, side effect
Zhai et al. [7]	24	CCMD-2-R	xiaoyao powder plus doxepin	doxepin	8		clinical effect, HAMD and TESS score, side effect
Zhang et al. [27]	59	CCMD-3	xiaoyao pill plus fluoxetine	fluoxetine	6		clinical effect, HAMD and TESS score, side effect
Zhang [28]	50	CCMD-3	xiaoyao pill plus fluoxetine	fluoxetine	6		HAMD score, side effect
Zhao and Jiang [29]	66	CCMD-3	modified xiaoyao powder plus amitriptyline	amitriptyline	12		symptoms, clinical effect, HAMD score, side effect
Zhou and Li [30]	90	CCMD-3	xiaoyao powder	fluoxetine	8		symptoms, clinical effect, HAMD and SDS score, side effect
Zhu [31]	60	CCMD-2	xiaoyao pill plus venlafaxine	venlafaxine	10		HAMD score, side effect
Zhu and Li [32]	60	CCMD-2	xiaoyao powder plus amitriptyline	amitriptyline	8		clinical effect, side effect

**Table 2:** Composition of formula.

ID	Formulation	Composition of formula
Du et al. [8]	pill	Chinese patent medicine

Huang and Gan [9]	decoction	Bupleuri 10 g, Paeoniae lactiflorae 15 g, Poriae cocos pararadicis 30 g, Atractylodis macrocephalae 15 g, Moutan radicis 10 g, Curcumae 10 g, Angelicae sinensis 15 g, Menthae haplocalycis 10 g, Tritici aestivi levis 30 g, Polygoni multiflory 30 g, Pseudostellariae heterophyllae 20 g. Menstrual period remove Moutan radicis, Pseudostellariae heterophyllae, Menthae haplocalycis, plus Ligustici chuanxiong 10 g, Persicae 10 g, Leonuri heterophylli 30 g, Guanzhong 10 g, typhae 10 g, Trogopteri seu pteromi 10 g; Follicular phase remove Moutan radicis, Menthae haplocalycis, plus Cervi 15 g, Epimedii 15 g, Chrysanthemi indici 15 g, Lycii 15 g; Luteal phase plus Gardeniae jasminoidis 10 g, Ligustri lucidi 20 g, Liquidam baris taiwanianae 15 g, Cyperi rotundi 10 g, Rhapontici seu echinops 10 g

Huo et al. [10]	decoction	Bupleuri 10 g, Paeoniae lactiflorae 12 g, Angelicae sinensis 12 g, Poriae cocos 20 g, Atractylodis macrocephalae 10 g, Moutan radicis 12 g, Gradeniae jasminodidis 10 g, Curcumae l2 g, Acori graminei 10 g, Fructus aurantii 10 g, Draconis 30 g, Ostreae 30 g, Polygalae tenuifoliae 12 g, Cizyphi spinosae 30 g, Tritici aestivi levis 30 g, Glycyrrhizae uralensis 10 g, Zizyphi jujubae 5. Blood stasis plus Ligustici chuanxiong 12 g, Salviae milgiorrhizae 20~30 g; Phelgm and dampness plus Citri reticulatae 10 g, Pinelliae ternatae 10 g; Yin deficiency plus Lilii 30 g, Anemarrhenae asphodeloibis 10 g; Qi deficiency remove Gradeniae jasminodidis, plus Pseudostellariae heterophyllae 15 g; Astriction plus Cannabis sativae 10 g or Radix et rhizome 10 g

Li et al. [11]	pill	Chinese patent medicine

Li et al. 2007 [12]	powder	Chinese patent medicine (bupleuri, angelicae sinensis, poriae cocos, atractylodis macrocephalae, gradeniae jasminodidis, moutan radicis)

Li et al. [13]	decoction	Bupleuri 15 g, Angelicae sinensis 15 g, Atractylodis macrocephalae 15 g, Paeoniae lactiflorae 10 g, Poriae cocos 10 g, Menthae haplocalycis 6 g, Glycyrrhizae uralensis 6 g

Liu and Chen [14]	decoction	Bupleuri 8 g, Gradeniae jasminodidis 6 g, Ligustici chuanxiong 6 g, Glycyrrhizae uralensis 6 g, Moutan radicis 10 g, Taeoniae rubrae 10 g, Atractylodis macrocephalae 10 g, Draconis 30 g, Ostreae 30 g, Poriae cocos 15 g. Liver qi stagnation and abdominal distention plus Aucklandiae lappae 10 g, Citri reticulatae 10 g, Cyperi rotundi 6 g; Insomnia plus Albizziae julibrissin 15 g, Polygoni multiflory 15 g, Poriae cocos pararadicis 15 g, Polygalae tenuifoliae 10 g; Cizyphi spinosae 10 g; Spleen and stomach deficiency plus Pseudostellariae heterophyllae 10 g, Citri reticulatae 10 g; Phlegm and dampness and no appetite plus Pinelliae ternatae 10 g, Bambusae in taeniis 10 g, Citri reticulatae 10 g, Amomi 6 g

Luo et al. [15]	powder	Bupleuri, Angelicae sinensis, Paeoniae lactiflorae, Poriae cocos, Atractylodis macrocephalae, Moutan radicis, Gradeniae jasminodidis

Ma [16]	pill	Chinese patent medicine

Wang et al. [17]	pill	Bupleuri, Astragali membranacei, Codonotsitis pilosulae, Angelicae sinensis, Paeoniae lactiflorae, Rehmanniae glutinosae, Artemisiae yinchenhao, Atractylodis macrocephalae, Poriae cocos, Aucklandiae lappae, Cizyphi spinosae, Polygalae tenuifoliae, Schisandrne chinensis, Acori graminei, Moutan radicis, Moschus, Menthae haplocalycis, Glycyrrhizae uralensis

Wang [18]	decoction	Bupleuri 12 g, Angelicae sinensis 20 g, Paeoniae lactiflorae 12 g, Atractylodis macrocephalae 12 g, Lilii 15 g, Albizziae julibrissin 15 g, Citri aurantii 10 g, Pinelliae ternatae 12 g, Gradeniae jasminodidis 10 g, Scutellariae baicalensis 10 g, Bambusae textillis 15 g, Curcumae 12 g, Acori graminei 12 g, Glycyrrhizae uralensis 6 g, Zizyphi jujubae 6

Wang [19]	decoction	Bupleuri 10 g, Paeoniae lactiflorae 12 g, Angelicae sinensis 12 g, Poriae cocos 20 g, Atractylodis macrocephalae 10 g, Moutan radicis 12 g, Gradeniae jasminodidis 10 g, Curcumae 12 g, Acori graminei 10 g, Fructus aurantii 10 g, Draconis 30 g, Ostreae 30 g, Polygalae tenuifoliae 12 g, Cizyphi spinosae 30 g, Tritici aestivi levis 30 g, Glycyrrhizae uralensis 10 g, Zizyphi jujubae 5. Blood stasis plus Ligustici chuanxiong 12 g, Salviae milgiorrhizae 20–30 g; Phlegm and dampness plus Citri reticulatae 10 g, Pinelliae ternatae 10 g; Yin deficiency plus Lilii 30 g, Anemarrhenae asphodeloibis 10 g; Qi deficiency remove Gradeniae jasminodidis, plus Pseudostellariae heterophyllae 15 g; Astriction plus Cannabis sativae 10 g or Radix et rhizome 10 g

Wang et al. [20]	pill	Chinese patent medicine

Wang and Liu [21]	decoction	Angelicae sinensis 10 g, Paeoniae lactiflorae 12 g, Bupleuri 12 g, Atractylodis macrocephalae 10 g, Poriae cocos 12 g, Zingiberis officinalis recens 10 g, Glycyrrhizae uralensis 10 g, Moutan radicis 10 g, Gradeniae jasminodidis 10 g

Wei et al. [22]	decoction	Qi stagnation and blood stasis plus Salviae milgiorrhizae, Cyperi rotundi, Linger strychnifoliae, Ligustici chuanxiong, Taeoniae rubrae; Qi stagnation leading to fire plus Salviae milgiorrhizae, Gardeniae jasminoidis; Qi and blood deficiency plus Codonotsitis pilosulae, Astragali membranacei, Rehmanniae glutinosae conquitae; Palpitation plus Cizyphi spinosae, Draconis, ostreae, Polygalae tenuifoliae, Biotae orientalin; Spleen and kidney deficiency plus Morindae officinalis, Curculiginis orchioidis, Epimedii, Zingberis officinalis, Aconiticarmichaeli praeparata, Corneum gigeriae galli

Xian et al. [23]	decoction	Angelicae sinensis, Paeoniae lactiflorae, Poriae cocos, Atractylodis macrocephalae, Menthae haplocalycis, Bupleuri, Zingiberis officinalis recens, Glycyrrhizae uralensis

Xiao [24]	decoction	Bupleuri 30 g, Menthae haplocalycis 12 g, Atractylodis macrocephalae 10 g, Poriae cocos 15 g, Moutan radicis 12 g, Gradeniae jasminodidis 12 g, Angelicae sinensis 12 g, Paeoniae lactiflorae 10 g, Zingiberis officinalis recens, Glycyrrhizae uralensis 6 g. Blood stasis Ligustici chuanxiong 12 g; Phelgm and dampness plus Citri reticulatae 10 g, Pinelliae ternatae 10 g; Yin deficiency plus Anemarrhenae asphodeloibis 10 g; Qi deficiency plus Pseudostellariae heterophyllae 15 g; Astriction plus Cannabis sativae 10 g or Radix et rhizome l0 g

Yang and Xie [25]	decoction	Bupleuri l5 g, Atractylodis macrocephalae l0 g, Paeoniae lactiflorae l0 g, Angelicae sinensis l0 g, Poliae cocos l0 g. Menthae haplocalycis 6 g, Glycyrrhizae uralensis 6 g. Qi stagnation leading to fire plus Gradeniae jasminodidis, Moutan radicis, Fructus aurantii; Heart and spleen deficiency plus Codonotsitis pilosulae, Astragali membranacei, Polygalae tenuifoliae, Dioscoreae oppositae; Phlegm and Qi stagnation plus Trichosanthis, Pinelliae ternatae, Madnoliae officinalis; Blood stasis plus Persicae, Salviae milgiorrhizae, Ligustici chuanxiong; Insomnia plus Cizyphi spinosae, Polygoni multiflori, Succinum

Yang et al. [26]	pill	Bupleuri, Angelicae sinensis, Paeoniae lactiflorae, Poriae cocos, Atractylodis macrocephalae, Moutan radicis, Gradeniae jasminodidis, Menthae haplocalycis, Glycyrrhizae uralensis

Zhai et al. [7]	pill	Chinese patent medicine

Zhang et al. [27]	pill	Bupleuri, Angelicae sinensis, Paeoniae lactiflorae, Atractylodis macrocephalae, Poriae cocos, Menthae haplocalycis, Zingiberis officinalis recens, Glycyrrhizae uralensis

Zhang [28]	pill	Chinese patent medicine

Zhao and Jiang [29]	decoction	Bupleuri 15 g, Angelicae sinensis 15 g, Paeoniae lactiflorae 15 g, Atractylodis macrocephalae 15 g, Poriae cocos 15 g, Moutan radicis 10 g, Gradeniae jasminodidis 10 g, Glycyrrhizae uralensis 10 g, Menthae haplocalycis 10 g, Zingiberis officinalis recens 10 g

Zhou and Li [30]	decoction	Bupleuri 12 g, Angelicae sinensis 12 g, Atractylodis macrocephalae 9 g, Poriae cocos 15 g, Paeoniae lactiflorae 12 g, Cizyphi spinosae 15 g, Salviae milgiorrhizae 30 g, Ligustici chuanxiong 12 g, Carthami tinctolii 12 g, Persicae 9 g, Gradeniae jasminodidis 12 g, Citri reticulatae viride 9 g, Glycyrrhizae uralensis 6 g

Zhu [31]	pill	Chinese patent medicine

Zhu and Li [32]	decoction	Heart and spleen deficiency plus Codonotsitis pilosulae, Astragali membranacei, Salviae milgiorrhizae, Polygalae tenuifoliae, Cizyphi spinosae, Asini. liver and kidney yin deficiency plus Draconis, Ostreae, Amydae sinensis, Asinilycii, Moutan radicis, Gradeniae jasminodidis. Liver qi stagnation plus Curcumae, Citri sarcodactylis, Citri reticulatae, Trichosanthis, Massa fermentata, Agastaches seu pogostemi, Eupatorii fortunei. Spleen and kidney yang deficiency plus Cinnamomi cassiae, Rehmanniae glutinosae conquitae, Corni officinalis, Schisandrne chinensis, Acori graminei

**Table 3:** Quality assessment of included randomized controlled trials.

Included trials	Sequence generation	Allocation concealment	Blinding of participants personnel and outcome assessors	Incomplete outcome data	Selective outcome reporting	Other sourcesof bias	Risk of bias
Du et al. [8]	Unclear	Unclear	Unclear	Yes	No	Unclear	High
Huang and Gan [9]	Unclear	Unclear	Unclear	No	No	Unclear	High
Huo et al. [10]	Table of random number	Unclear	Unclear	No	No	Unclear	Unclear
J. Li et al. [11]	Unclear	Unclear	Unclear	Yes	No	Unclear	Unclear
Liu and Chen [14]	Unclear	Unclear	Double blind	Yes	No	Unclear	Unclear
Li et al. [13]	Unclear	Unclear	Unclear	Yes	No	Unclear	High
Liu and Chen [14]	Unclear	Unclear	Unclear	No	No	Unclear	High
Luo et al. [15]	Table of random number	Unclear	Double blind	Yes	No	Unclear	Unclear
Ma [16]	Table of random number	Unclear	Unclear	Yes	Yes	Unclear	Unclear
Wang et al. [17]	Unclear	Unclear	Unclear	No	No	Unclear	High
Wang [18]	Unclear	Unclear	Unclear	No	No	Unclear	High
Wang [19]	Unclear	Unclear	Unclear	No	No	Unclear	High
Wang et al. [20]	Unclear	Unclear	Unclear	No	Yes	Unclear	High
Wang and Liu [21]	Unclear	Unclear	Unclear	No	Yes	Unclear	High
Wei et al. [22]	Unclear	Unclear	Unclear	No	Yes	Unclear	High
Xian et al. [23]	Table of random number	Yes	Unclear	No	Yes	Unclear	Unclear
Xiao [24]	Unclear	Unclear	Unclear	No	No	Unclear	High
Yang and Xie [25]	Unclear	Unclear	Unclear	No	Yes	Unclear	High
Yang et al. [26]	Table of random number	Unclear	Unclear	No	No	Unclear	Unclear
Zhai et al. [7]	Unclear	Unclear	Unclear	No	No	Unclear	High
Zhang et al. [27]	Unclear	Unclear	Single-blind	No	No	Unclear	Unclear
Zhang [28]	Unclear	Unclear	Unclear	No	No	Unclear	High
Zhao and Jiang [29]	Unclear	Unclear	Unclear	No	No	Unclear	High
Zhou and Li [30]	Unclear	Unclear	Unclear	No	No	Unclear	High
Zhu [31]	Unclear	Unclear	Unclear	No	No	Unclear	High
Zhu and Li [32]	Unclear	Unclear	Unclear	No	No	Unclear	High

**Table 4:** Analyses of clinical comprehensive effect.

Trials		Intervention (*n*/*N*)	Control (*n*/*N*)	RR [95% CI]	*P *Value
*Xiaoyao powder versus antidepressants*					
Xiaoyao powder versus amitriptyline	1	41/42	25/27	1.05 [0.94, 1.18]	0.37
Danzhi Xiaoyao powder versus maprotiline	1	28/32	30/31	0.90 [0.78, 1.05]	0.18
Guipi Xiaoyao powder versus venlafaxine	1	94/100	86/100	1.09 [1.00, 1.20]	0.06
Modified Xiaoyao powder versus amitriptyline	1	29/30	28/30	1.04 [0.92, 1.16]	0.55
Danzyhih Xiaoyao decoction versus fluoxetine	1	29/34	22/27	1.05 [0.83, 1.31]	0.69
Xiaoyao powder versus fluoxetine	2	66/75	61/75	1.08 [0.94, 1.24]	0.25

*Meta-Analysis*	7	287/313	252/290	1.05 [1.00, 1.11]	0.07

*Xioayao powder plus antidepressants versus antidepressants*					
Xiaoyao pills/powder plus amitriptyline versus amitriptyline	2	56/60	45/60	1.24 [1.06, 1.46]	0.008
Danzhi Xiaoyao decoction/powder plus fluoxetine versus fluoxetine	2	51/66	38/66	1.34 [1.05, 1.72]	0.02
Xiaoyao pills plus imipramine versus imipramine	1	26/30	25/29	1.01 [0.82, 1.23]	0.96
Xiaoyao pills plus fluoxetine versus fluoxetine	2	56/71	54/66	0.97 [0.82, 1.14]	0.69
Danzhi Xiaoyao powder plus amitriptyline versus amitriptyline	1	38/40	28/38	1.29 [1.05, 1.58]	0.01
Xiaoyao pills plus citalopram versus citalopram	1	28/30	28/30	1.00 [0.87, 1.14]	1.00
Xiaoyao pills plus doxepin versus fluoxetine	1	32/35	29/33	1.04 [0.88, 1.22]	0.63
Danzhi Xiaoyao powder plus clomipramine versus clomipramine	1	30/33	25/33	1.20 [0.96, 1.50]	0.11
Modified Xiaoyao pills plus amitriptyline versus fluoxetine	1	29/32	30/32	0.97 [0.84, 1.12]	0.64
Modified Xiaoyao powder plus amitriptyline versus amitriptyline	1	31/33	30/33	1.03 [0.90, 1.19]	0.64
Xiaoyao powder plus doxepin versus doxepin	1	9/12	10/12	0.90 [0.60, 1.36]	0.62

*Meta-Analysis*	14	386/442	342/432	1.10 [1.04, 1.17]	0.001

**Table 5:** Analyses of score of HAMD.

Trials		WMD [95% CI]	*P *value
*Xiaoyao powder versus antidepressants*			
Danzhi Xiaoyao powder versus maprotiline	2	2.39 [−0.55, 5.33]	0.11
Modified Xiaoyao powder versus amitriptyline	1	1.11 [−0.21, 2.43]	0.10
Xiaoyao powder versus fluoxetine	1	−3.30 [−6.07, −0.53]	0.02

*Meta-Analysis *[*FEM*]	4	0.59 [−0.51, 1.70]	0.29

*Meta-Analysis* [*REM*]	4	0.43 [−2.14, 2.99]	0.74

*Xiaoyao powder plus antidepressants versus antidepressants*			
Xiaoyao pills plus fluoxetine versus amitriptyline	1	0.06 [−1.50, 1.62]	0.94
Xiaoyao pills plus fluoxetine versus paroxetine	1	1.88 [0.20, 3.56]	0.03
Danzhi Xiaoyao decoction/powder plus fluoxetine versus fluoxetine	2	−0.18 [−0.49, 0.13]	0.25
Xiaoyao pills plus imipramine versus imipramine	1	−0.10 [−2.35, 2.15]	0.93
Xiaoyao pills plus fluoxetine versus fluoxetine	3	−1.41 [−2.17, −0.65]	0.0003
Danzhi Xiaoyao powder plus amitriptyline versus amitriptyline	1	−5.84 [−8.76, −2.92]	<0.0001
Xiaoyao pills plus citalopram versus citalopram	1	−0.30 [−2.06, 1.46]	0.74
Xiaoyao pills plus doxepin versus fluoxetine	1	−0.88 [−1.38, −0.38]	0.0006
Modified Xiaoyao pills plus amitriptyline versus fluoxetine	1	0.17 [−1.29, 1.63]	0.82
Xiaoyao powder plus doxepin versus doxepin	1	−0.80 [−4.84, 3.24]	0.70
Modified Xiaoyao powder plus amitriptyline versus amitriptyline	1	−0.76 [−1.16, −0.36]	0.0002

*Meta-Analysis* [*FEM*]	14	−0.51 [−0.71, −0.31]	<0.00001

*Meta-Analysis* [*REM*]	14	−0.69 [−1.25, −0.13]	0.02

FEM: fixed effects model, REM: random effects model.

**Table 6:** Analyses of score of SDS.

Trials		WMD [95% CI]	*P *value
*Xiaoyao powder versus antidepressants*			
Danzhi Xiaoyao powder versus maprotiline	1	−1.19 [−10.84, 8.46]	0.81
Guipi Xiaoyao powder versus venlafaxine	1	−5.00 [−7.07, −2.93]	<0.00001
Danzyhih Xiaoyao decoction versus fluoxetine	1	−5.14 [−9.54, −0.74]	0.02
Xiaoyao powder versus fluoxetine	1	−1.70 [−4.59, 1.19]	0.25

*Meta-Analysis* [*FEM*]	4	−3.97 [−5.52, −2.41]	<0.00001

*Xioayao powder plus antidepressants versus antidepressants*			
Xiaoyao pills plus fluoxetine versus fluoxetine	1	−3.60 [−4.65, −2.55]	<0.00001
